# Arginase attenuates inhibitory nonadrenergic noncholinergic nerve-induced nitric oxide generation and airway smooth muscle relaxation

**DOI:** 10.1186/1465-9921-6-23

**Published:** 2005-03-04

**Authors:** Harm Maarsingh, Marieke A Tio, Johan Zaagsma, Herman Meurs

**Affiliations:** 1 Department of Molecular Pharmacology, University Centre for Pharmacy, University of Groningen, Antonius Deusinglaan 1, 9713 AV Groningen, The Netherlands

## Abstract

**Background:**

Recent evidence suggests that endogenous arginase activity potentiates airway responsiveness to methacholine by attenuation of agonist-induced nitric oxide (NO) production, presumably by competition with epithelial constitutive NO synthase for the common substrate, L-arginine. Using guinea pig tracheal open-ring preparations, we now investigated the involvement of arginase in the modulation of neuronal nitric oxide synthase (nNOS)-mediated relaxation induced by inhibitory nonadrenergic noncholinergic (iNANC) nerve stimulation.

**Methods:**

Electrical field stimulation (EFS; 150 mA, 4 ms, 4 s, 0.5 – 16 Hz)-induced relaxation was measured in tracheal preparations precontracted to 30% with histamine, in the presence of 1 μM atropine and 3 μM indomethacin. The contribution of NO to the EFS-induced relaxation was assessed by the nonselective NOS inhibitor L-NNA (0.1 mM), while the involvement of arginase activity in the regulation of EFS-induced NO production and relaxation was investigated by the effect of the specific arginase inhibitor nor-NOHA (10 μM). Furthermore, the role of substrate availability to nNOS in EFS-induced relaxation was measured in the presence of various concentrations of exogenous L-arginine.

**Results:**

EFS induced a frequency-dependent relaxation, ranging from 6.6 ± 0.8% at 0.5 Hz to 74.6 ± 1.2% at 16 Hz, which was inhibited with the NOS inhibitor L-NNA by 78.0 ± 10.5% at 0.5 Hz to 26.7 ± 7.7% at 8 Hz (P < 0.01 all). In contrast, the arginase inhibitor nor-NOHA increased EFS-induced relaxation by 3.3 ± 1.2-fold at 0.5 Hz to 1.2 ± 0.1-fold at 4 Hz (P < 0.05 all), which was reversed by L-NNA to the level of control airways in the presence of L-NNA (P < 0.01 all). Similar to nor-NOHA, exogenous L-arginine increased EFS-induced airway relaxation (P < 0.05 all).

**Conclusion:**

The results indicate that endogenous arginase activity attenuates iNANC nerve-mediated airway relaxation by inhibition of NO generation, presumably by limiting L-arginine availability to nNOS.

## Background

The inhibitory nonadrenergic noncholinergic (iNANC) nervous system is the most effective bronchodilating neural pathway of the airways. Inhibition of nitric oxide synthase (NOS) markedly reduces the iNANC relaxation of both guinea pigs [1-3] and human airways [4,5], indicating that nitric oxide (NO) is a major neurotransmitter of the iNANC system. In addition, vasoactive intestinal polypeptide (VIP) has been implicated in iNANC relaxation [6,7], and colocalization of NOS and VIP has been demonstrated both in guinea pig [8] and in human airway nerves [9].

NO is generated by a family of NOS isoforms that utilize the semi-essential amino acid L-arginine, oxygen and NADPH as substrates to produce NO and L-citrulline [10]. Three isoforms of NOS have been identified: neuronal NOS (nNOS), endothelial NOS (eNOS) and inducible NOS (iNOS). In the airways, the constitutive NOS (cNOS) isoforms are mainly expressed in the iNANC neurons (nNOS), the endothelium (eNOS) and the epithelium (nNOS and eNOS), whereas iNOS, which is induced by proinflammatory cytokines during airway inflammation, is mainly expressed in macrophages and epithelial cells [11].

Another L-arginine metabolizing enzyme is arginase, which hydrolyzes L-arginine to L-ornithine and urea. Arginase is classically considered to be an enzyme of the urea cycle in the liver, but also occurs in extrahepatic tissues, including the lung [12,13]. Two distinct isoforms of arginase have been identified in mammals: arginase I, a cytosolic enzyme, mainly expressed in the liver, and arginase II, a mitochondrial enzyme, which is mainly expressed in extrahepatic tissues [13]. Extrahepatic arginase has been implicated in the regulation of NO synthesis by limiting the availability of intracellular L-arginine for NOS [12-15]. In addition, arginase might be involved in cell growth and tissue repair via the production of L-ornithine, a precursor of polyamines and proline [13]. Both arginase isoforms are constitutively expressed in the airways, particularly in the bronchial epithelium and in fibroblasts from peribronchial connective tissue [12]. Using a perfused guinea pig tracheal tube preparation, we have previously demonstrated that endogenous arginase activity is functionally involved in the regulation of airway smooth muscle tone [16]. Endogenous arginase potentiates methacholine-induced airway constriction by diminishing agonist-induced NO production, by competition with epithelial cNOS for the common substrate, L-arginine [16]. Previous studies had demonstrated that L-arginine availability is indeed a limiting factor for agonist-induced NO-production and airway relaxation [17].

A role for arginase in the iNANC system has been found in internal anal sphincter [18] and penile corpus cavernosum [19,20]. Thus, arginase inhibition increased electrical field stimulation (EFS)-induced relaxation of these preparations, indicating that endogenous arginase activity attenuates nNOS-mediated NANC relaxation.

The role of endogenous arginase in the regulation of iNANC-derived NO generation in the airways has not yet been investigated. In the present study, we demonstrated that endogenous arginase activity and L-arginine availability are importantly involved in the modulation of iNANC nerve-mediated NO-production and relaxation of guinea pig tracheal smooth muscle.

## Methods

### Animals

Male specific pathogen free HsdPoc:Dunkin Hartley guinea pigs (Harlan Heathfield, UK), weighing 500 – 800 g, were used in this study. The animals were group-housed in individual cages in climate-controlled animal quarters and given water and food *ad libitum*, while a 12-h on/12-h off light cycle was maintained.

All protocols described in this study were approved by the University of Groningen Committee for Animal Experimentation.

### Tissue preparation

The guinea pigs were sacrificed by a sharp blow on the head. After exsanguination, the trachea was removed from the larynx to the bronchi and rapidly placed in a Krebs-Henseleit (KH) buffer solution of 37°C, gassed with 95% O_2 _and 5% CO_2_. The composition of the KH-solution in mM was: NaCl 117.50; KCl 5.60; MgSO_4 _1.18; CaCl_2 _2.50; NaH_2_PO_4 _1.28; NaHCO_3 _25.0 and D-glucose 5.50; pH 7.4. The trachea was prepared free of serosal connective tissue. Twelve single proximal tracheal open-ring preparations were mounted for isotonic recording (0.3 g preload) between two parallel platinum point-electrodes in water-jacketed (37°C) organ baths containing 20.0 ml of gassed KH-solution and indomethacin (3 μM), which remained present during the whole experiment to eliminate any influence of prostanoids.

### Electrical field stimulation-induced relaxation experiments

After a 30 min equilibration period, tracheal preparations were relaxed with isoprenaline (0.1 μM) to establish basal tone. After a washout period of 30 min with three washes with fresh KH solution, maximal contraction of the tracheal preparations to histamine was determined with cumulative additions of the agonist (0.1, 1, 10 and 100 μM). After washout (30 min), the tracheal preparations were precontracted with histamine to 30% of the maximal histamine-induced tone in the presence of atropine (1 μM) to prevent EFS-induced cholinergic airway contraction. On the plateau, biphasic EFS (150 mA, 4 ms, 4 s, 0.5 – 16 Hz) was applied and frequency response curves (0.5 – 16 Hz in doubling steps) were recorded. Per preparation, one frequency response curve was performed. When used, the nonselective NOS inhibitor N^ω^-nitro-L-arginine (L-NNA; 100 μM), the specific arginase inhibitor N^ω^-hydroxy-nor-L-arginine (nor-NOHA; 10 μM), a combination of both inhibitors, or L-arginine (0.3, 1.0 or 5.0 mM) were applied 30 min prior to histamine-addition. In line with previous observations [21], neither the NOS inhibitor, nor the arginase inhibitor and L-arginine affected agonist-induced tone in the open-ring preparations. All measurements were performed in triplicate. After the final EFS-induced relaxation, followed by washout, isoprenaline (10 μM) was added to establish basal tone.

### Data analysis

All individual relaxations elicited by EFS were estimated as peak height of the EFS-induced response, and were expressed as a percentage of maximal relaxation as established in the presence of isoprenaline. The contribution of NO to the EFS-induced relaxation was determined by the effect of the NOS inhibitor L-NNA. Similarly, the role of arginase activity in the modulation of EFS-induced airway relaxation was determined by the effect of the arginase inhibitor nor-NOHA. The role of substrate availability in EFS-induced airway relaxation was assessed by measuring the responses in the presence of various concentrations of exogenous L-arginine.

All data are expressed as means ± s.e.m. Statistical significance of differences was evaluated using a paired or unpaired two-tailed Student's t-test as appropriate, and significance was accepted when *P *< 0.05.

### Chemicals

Histamine dihydrochloride, indomethacin, atropine sulphate, N^ω^-nitro-L-arginine, (-)-isoprenaline hydrochloride and L-arginine hydrochloride were obtained from Sigma Chemical Co. (St. Louis, MO, USA). N^ω^-hydroxy-nor-L-arginine was kindly provided by Dr J.-L. Boucher (Université Paris V).

## Results

**Figure 1:**
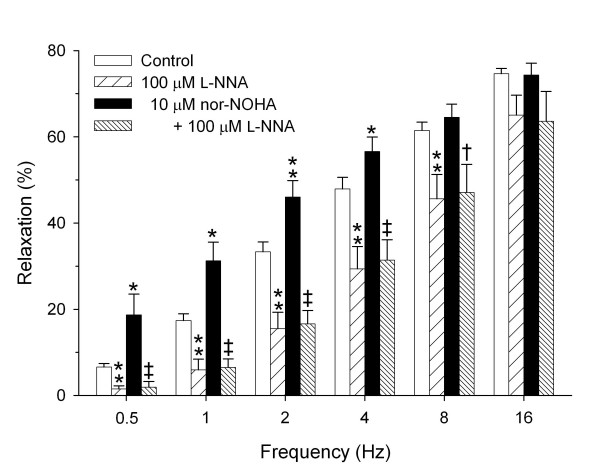
**Role of NO and arginase in iNANC nerve-induced relaxation of guinea pig tracheal smooth muscle. **Electrical field stimulation-induced relaxation of precontracted guinea pig tracheal open-ring preparations in the absence and presence of the NOS inhibitor L-NNA (100 μM), the arginase inhibitor nor-NOHA (10 μM) or a combination of both inhibitors. Results are means ± s.e.m. of 8 experiments. *P < 0.05 and **P < 0.01 compared to control, ^†^*P *< 0.05 and ^‡^*P *< 0.01 compared to nor-NOHA-treated.

In guinea pig tracheal open-ring preparations, EFS induced a frequency-dependent relaxation of histamine-induced tone ranging from 6.6 ± 0.8% at 0.5 Hz to 74.6 ± 1.2% at 16 Hz. Incubation with the NOS inhibitor L-NNA caused a significant inhibition of the EFS-induced relaxation at 0.5 to 8 Hz, particularly at the lower frequencies. The effect of L-NNA ranged from 78.0 ± 10.5% inhibition at 0.5 Hz to 26.7 ± 7.7% inhibition at 8 Hz (*P *< 0.01 all; Fig. 1).

In contrast, incubation with the arginase inhibitor nor-NOHA significantly enhanced EFS-induced relaxation by 3.3 ± 1.2-fold at 0.5 Hz to 1.2 ± 0.1-fold at 4 Hz (*P *< 0.05 all; Fig. 1), that is, at the frequencies most sensitive to L-NNA. The increased relaxation in the presence of nor-NOHA was fully reverted by L-NNA (*P *< 0.05 all), to the level of control preparations in the presence of L-NNA alone (Fig. 1).

**Figure 2:**
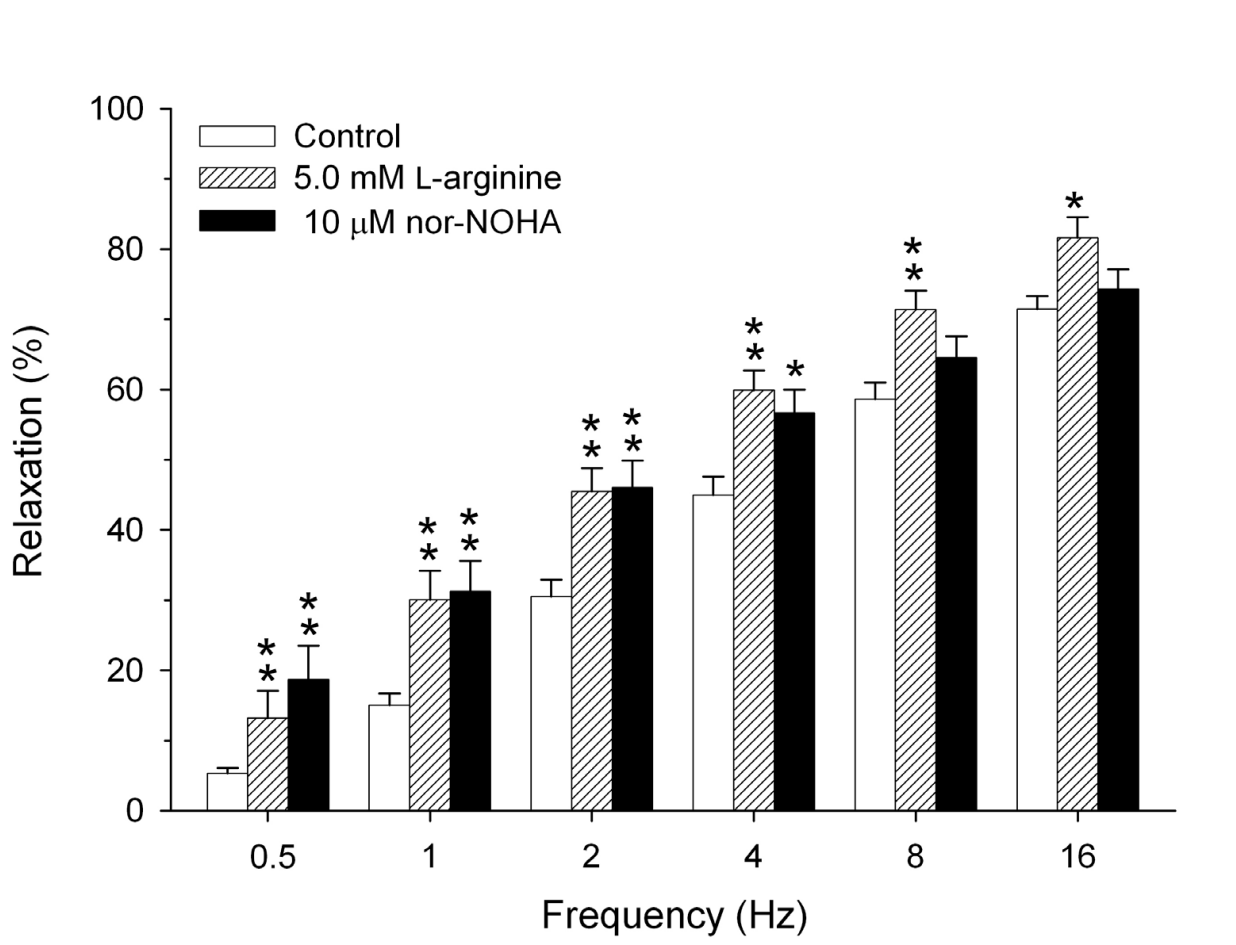
**Role of L-arginine availability and arginase in iNANC nerve-induced relaxation of guinea pig tracheal smooth muscle. **Electrical field stimulation-induced relaxation of precontracted guinea pig tracheal open-ring preparations in the absence and presence of exogenous L-arginine (5.0 mM) or the arginase inhibitor nor-NOHA (10 μM). Results are means ± s.e.m. of 5–13 experiments. *P < 0.05 and **P < 0.01 compared to control.

Incubation with L-arginine caused a dose-dependent increase of total EFS-induced relaxation, which was maximal at 5.0 mM L-arginine (data not shown). In the presence of 5.0 mM L-arginine, a significant increase in EFS-induced relaxation was observed at all frequencies compared to untreated preparations (*P *< 0.05 all, Fig. 2). At the lower frequencies, this increase was similar to the increase in EFS-induced relaxation observed after incubation with nor-NOHA (Fig. 2).

## Discussion

Using perfused tracheal preparations, we have previously demonstrated that endogenous arginase activity is involved in the regulation of agonist-induced airway constriction by inhibition of NO production, presumably by competition with cNOS for L-arginine [16]. In the present study, we demonstrated that endogenous arginase activity is also involved in the regulation of iNANC nerve-mediated airway smooth muscle relaxation.

In line with previous observations [1], it was demonstrated that the NOS inhibitor L-NNA inhibited EFS-induced iNANC relaxation of guinea pig tracheal preparations. This inhibition was most pronounced at the lower frequencies, indicating a prominent role of nNOS-derived NO at these frequencies. By contrast, inhibition of arginase activity by nor-NOHA caused a considerable (up to 3.3-fold) increase in EFS-induced relaxation at low frequencies, indicating that endogenous arginase activity restricts iNANC nerve-mediated airway smooth muscle relaxation. The increased relaxation after arginase inhibition was completely reverted by L-NNA, clearly indicating that arginase activity attenuates iNANC nerve-mediated airway smooth muscle relaxation by limiting NO production, presumably by competition with nNOS for their common substrate, L-arginine.

The observation that exogenous L-arginine significantly enhanced the EFS-induced airway smooth muscle relaxation confirms that L-arginine is indeed a limiting factor in EFS-induced, NO-mediated airway smooth muscle relaxation under basal conditions. Remarkably, the effect of nor-NOHA was similar to that observed in the presence of the maximally effective L-arginine concentration, indicating that endogenous arginase activity is a major factor in regulating the neural NO-mediated airway smooth muscle relaxation.

Recently, we discovered that increased arginase activity is importantly involved in the pathophysiology of asthma by contributing to the allergen-induced NO-deficiency and subsequent airway hyperresponsiveness to methacholine after the early asthmatic reaction, by limiting the availability of L-arginine for cNOS to produce bronchodilating NO [22]. Arginase activity as well as expression was also considerably increased in two mouse models of allergic asthma, irrespective whether the animals were challenged with ovalbumin or with *Aspergillus fumigatus *[23]. Moreover, enhanced mRNA or protein expression of arginase I was observed in human asthmatic lung tissue, particularly in inflammatory cells and in the airway epithelium [23], while increased arginase activity was measured in asthmatic serum [24]. In guinea pig tracheal strips, it has previously been demonstrated that EFS-induced iNANC relaxation is reduced after ovalbumin-challenge, due to a deficiency of iNANC nerve-derived NO [25]. Thus, it is tempting to speculate that increased arginase activity could similarly be involved in allergen-induced reduced iNANC activity.

A role for arginase by restricting the L-arginine availability for nNOS in iNANC nerves has also been proposed in the pathophysiology of erectile dysfunction [19]. In support, increased expression and activity of arginase II contributing to reduced NO production has been demonstrated in diabetic cavernosal tissue [26]. Neuronal arginase may also be involved in gastrointestinal motility disorders, by reducing nNOS-mediated iNANC relaxation in the internal anal sphincter [18].

## Conclusion

This is the first demonstration that endogenous arginase activity is functionally involved in iNANC nerve activity in the airways, by attenuating the generation of nNOS-derived NO. Disturbance of this novel regulation mechanism of airway responsiveness might be involved in the pathophysiology of allergic asthma.

## Abbreviations

cNOS, constitutive nitric oxide synthase; EFS, electrical field stimulation; eNOS, endothelial nitric oxide synthase; iNANC, inhibitory nonadrenergic noncholinergic; iNOS, inducible nitric oxide synthase; KH, Krebs-Henseleit; L-NNA, N^ω^-nitro-L-arginine; NADPH, nicotinamide adenine dinucleotide phosphate; nNOS, neuronal nitric oxide synthase; nor-NOHA, N^ω^-hydroxy-nor-L-arginine; VIP, vasoactive intestinal polypeptide

## Competing interests

The authors declare that they have no competing interests.

## Authors' contributions

HMa designed and coordinated the study, performed a major part of the experiments, performed the statistical analysis and drafted the manuscript. MAT assisted substantially in performing the experiments. JZ participated in the design of the study, interpretation of results and final revision of the manuscript. HMe conceived of the study, participated in its design and direction, as well as in preparing the manuscript. All authors read and approved the final manuscript.

## References

[B1] Tucker JF, Brave SR, Charalambous L, Hobbs AJ, Gibson A (1990). L-NG-nitro arginine inhibits non-adrenergic, non-cholinergic relaxations of guinea-pig isolated tracheal smooth muscle. Br J Pharmacol.

[B2] Belvisi MG, Stretton D, Barnes PJ (1991). Nitric oxide as an endogenous modulator of cholinergic neurotransmission in guinea-pig airways. Eur J Pharmacol.

[B3] Li CG, Rand MJ (1991). Evidence that part of the NANC relaxant response of guinea-pig trachea to electrical field stimulation is mediated by nitric oxide. Br J Pharmacol.

[B4] Belvisi MG, Stretton CD, Yacoub M, Barnes PJ (1992). Nitric oxide is the endogenous neurotransmitter of bronchodilator nerves in humans. Eur J Pharmacol.

[B5] Ellis JL, Undem BJ (1992). Inhibition by L-NG-nitro-L-arginine of nonadrenergic-noncholinergic-mediated relaxations of human isolated central and peripheral airway. Am Rev Respir Dis.

[B6] Ellis JL, Farmer SG (1989). Effects of peptidases on non-adrenergic, non-cholinergic inhibitory responses of tracheal smooth muscle: a comparison with effects on VIP- and PHI-induced relaxation. Br J Pharmacol.

[B7] Ellis JL, Farmer SG (1989). The effects of vasoactive intestinal peptide (VIP) antagonists, and VIP and peptide histidine isoleucine antisera on non-adrenergic, non-cholinergic relaxations of tracheal smooth muscle. Br J Pharmacol.

[B8] Shimosegawa T, Toyota T (1994). NADPH-diaphorase activity as a marker for nitric oxide synthase in neurons of the guinea pig respiratory tract. Am J Respir Crit Care Med.

[B9] Fischer A, Hoffmann B (1996). Nitric oxide synthase in neurons and nerve fibers of lower airways and in vagal sensory ganglia of man. Correlation with neuropeptides. Am J Respir Crit Care Med.

[B10] Moncada S, Palmer RM, Higgs EA (1989). Biosynthesis of nitric oxide from L-arginine. A pathway for the regulation of cell function and communication. Biochem Pharmacol.

[B11] Ricciardolo FL, Sterk PJ, Gaston B, Folkerts G (2004). Nitric oxide in health and disease of the respiratory system. Physiol Rev.

[B12] Que LG, Kantrow SP, Jenkinson CP, Piantadosi CA, Huang YC (1998). Induction of arginase isoforms in the lung during hyperoxia. Am J Physiol.

[B13] Wu G, Morris SM (1998). Arginine metabolism: nitric oxide and beyond. Biochem J.

[B14] Hey C, Boucher JL, Vadon-Le GS, Ketterer G, Wessler I, Racke K (1997). Inhibition of arginase in rat and rabbit alveolar macrophages by N omega-hydroxy-D,L-indospicine, effects on L-arginine utilization by nitric oxide synthase. Br J Pharmacol.

[B15] Boucher JL, Moali C, Tenu JP (1999). Nitric oxide biosynthesis, nitric oxide synthase inhibitors and arginase competition for L-arginine utilization. Cell Mol Life Sci.

[B16] Meurs H, Hamer MA, Pethe S, Vadon-Le GS, Boucher JL, Zaagsma J (2000). Modulation of cholinergic airway reactivity and nitric oxide production by endogenous arginase activity. Br J Pharmacol.

[B17] De Boer J, Duyvendak M, Schuurman FE, Pouw FM, Zaagsma J, Meurs H (1999). Role of L-arginine in the deficiency of nitric oxide and airway hyperreactivity after the allergen-induced early asthmatic reaction in guinea-pigs. Br J Pharmacol.

[B18] Baggio R, Emig FA, Christianson DW, Ash DE, Chakder S, Rattan S (1999). Biochemical and functional profile of a newly developed potent and isozyme-selective arginase inhibitor. J Pharmacol Exp Ther.

[B19] Cox JD, Kim NN, Traish AM, Christianson DW (1999). Arginase-boronic acid complex highlights a physiological role in erectile function. Nat Struct Biol.

[B20] Kim NN, Cox JD, Baggio RF, Emig FA, Mistry SK, Harper SL, Speicher DW, Morris SMJ, Ash DE, Traish A, Christianson DW (2001). Probing erectile function: S-(2-boronoethyl)-L-cysteine binds to arginase as a transition state analogue and enhances smooth muscle relaxation in human penile corpus cavernosum. Biochemistry.

[B21] De Boer J, Meurs H, Coers W, Koopal M, Bottone AE, Visser AC, Timens W, Zaagsma J (1996). Deficiency of nitric oxide in allergen-induced airway hyperreactivity to contractile agonists after the early asthmatic reaction: an ex vivo study. Br J Pharmacol.

[B22] Meurs H, McKay S, Maarsingh H, Hamer MA, Macic L, Molendijk N, Zaagsma J (2002). Increased arginase activity underlies allergen-induced deficiency of cNOS-derived nitric oxide and airway hyperresponsiveness. Br J Pharmacol.

[B23] Zimmermann N, King NE, Laporte J, Yang M, Mishra A, Pope SM, Muntel EE, Witte DP, Pegg AA, Foster PS, Hamid Q, Rothenberg ME (2003). Dissection of experimental asthma with DNA microarray analysis identifies arginase in asthma pathogenesis. J Clin Invest.

[B24] Morris CR, Poljakovic M, Lavrisha L, Machado L, Kuypers FA, Morris SM (2004). Decreased arginine bioavailability and increased serum arginase activity in asthma. Am J Respir Crit Care Med.

[B25] Miura M, Yamauchi H, Ichinose M, Ohuchi Y, Kageyama N, Tomaki M, Endoh N, Shirato K (1997). Impairment of neural nitric oxide-mediated relaxation after antigen exposure in guinea pig airways in vitro. Am J Respir Crit Care Med.

[B26] Bivalacqua TJ, Hellstrom WJ, Kadowitz PJ, Champion HC (2001). Increased expression of arginase II in human diabetic corpus cavernosum: in diabetic-associated erectile dysfunction. Biochem Biophys Res Commun.

